# Bloodstream infection in cancer patients; susceptibility profiles of the isolated pathogens, at Khartoum Oncology Hospital, Sudan

**DOI:** 10.4314/ahs.v22i4.10

**Published:** 2022-12

**Authors:** Omeima Mohammed Zain, Mohimmen Yousif Elsayed, Sozan M Abdelkhalig, Manal Abdelaziz, Safaa Yahia Ibrahim, Tahane Bashir, Yassir Hamadalnil

**Affiliations:** 1 Department of Microbiology, Khartoum oncology hospital, Khartoum, Sudan; 2 Department of Microbiology, Faculty of Medicine, AlMaarefa Uinversity, Riyadh, Saudi Arabia; 3 Department of Microbiology, Alzaeem Alazhari university, Khartoum, Sudan; 4 Department of Clinical Pharmacy, Omdurman Islamic University, Khartoum, Sudan; 5 Faculty of Medicine, Red Sea University, Port Sudan, Sudan; 6 Department of Microbiology, Faculty of Medicine, Nile University, Khartoum, Sudan; 6 Pathology Department - Ibra Hospital – Ministry of Health – Oman

**Keywords:** Blood stream infection, malignancy, antibiotics sensitivity, Sudan

## Abstract

**Introduction:**

Bloodstream infection is one of the major causes of mortality in patients with malignancies. This study aimed to determine the local profile of blood culture isolates and their antibiotic sensitivities in febrile neutropenic cancer patients and to decide if any modifications to antibiotics policies are necessary.

**Methods:**

This is a cross-sectional study conducted between the first of October to the end of December 2018 at Khartoum Oncology Hospital, Sudan. Blood samples from febrile neutropenic patients were collected for culture. Isolates were identified, and their antimicrobial susceptibility was determined by standard laboratory procedures.

**Results:**

Bloodstream infections were confirmed in 12 % (n = 69/569) of total blood cultures. Gram negative bacilli were the dominant causative agents (63.8%) while (36.2%) of infections were caused by gram positive cocci. Escherichia coli was the most common isolate (30.4%).

The proportions of resistance among gram negative bacilli were high for cefuroxime, amoxicillin/clavulanic acid, Ceftazidime, and ceftriaxone. Extended-spectrum β-lactamase producing isolates were identified in 34.1% of the positive cultures. Gram positive cocci showed high resistance to tetracycline, penicillin and erythromycin but were completely sensitive to vancomycin and gentamicin. Most of Staphylococcus aureus isolates were methicillin resistant.

**Conclusion:**

Gram negative bacilli were the predominant etiologic agents of bloodstream infections in our patients. Both Gram-positive and Gram-negative bacteria showed high levels of resistance for most of the common antibiotics used for empiric treatment. Regular surveillance to study bacterial resistance patterns must be conducted to modify antibiotics stewardship in our institution.

## Introduction

Patients with cancer usually have weak immune system due to several reasons including invasion of bone marrow, immunosuppressive anticancer chemotherapy and co-morbid conditions such as malnutrition. This immunodeficiency status exposes them to many infections mainly blood stream infection (BSI) 1. BSI often result in prolong hospitalization, delay of chemotherapy and increase in the cost of treatment, leading to significant increase in morbidity and mortality rates 2. Mortality due to BSI in cancer patients is reported at 11–40 % 3. BSI are usually acquired in the hospitals and health care centres (nosocomial) but can also be community-acquired infections 3. The risk of BSI in these patients is high due to the frequent interaction with health care setting for treatment. Risk factors include frequent hospitalization, insertion of vascular access devices, and the use of broad-spectrum antibiotics 1,4,5. Using of empirical antibiotics therapy resulted in emergence of multidrug-resistant (MDR) organisms and has become a major health problem worldwide, especially in cancer patients, and had led to increased morbidity and mortality among patients with BSI 6. Empirical antibacterial therapy in cancer patients should be chosen depending on types of isolates, their sensitivity patterns and the suspected sources of infection to decrease the incidence of antimicrobial resistant 7. The pattern of bacterial organisms causing bloodstream infection in cancer patients constantly changes and the emergence of resistant bacteria has further complicated the problem. Changes in the use of antibiotics prophylaxis shifted the causative agents from gram positive to gram negative bacteria with marked increase in antibiotics resistance 8. The data of infection in cancer patients in Sudan is very limited. Therefore, we conducted this study to investigate the BSI in cancer patient at Khartoum Oncology hospital in Khartoum, which is the largest oncology centre in Sudan, to determine the spectrum of the causative pathogens and their antimicrobial susceptibility patterns.

## Methods

### Study setting, and design

This cross-sectional, hospital-based study was conducted in Khartoum Oncology Hospital in Khartoum, Sudan. This 150-bed hospital is the largest oncology centre in Sudan and patients are referred to it from all cities in the country. Ethical approval was obtained from the ethical committee of the hospital and from Sudan Medical Specialization Board. Verbal consent was obtained from all the patients.

The study was conducted between the period of the first of October and the end of December 2018.

### Study population

Blood samples were collected from 569 cancer patients, who had a fever of ≥38 °C and a neutrophil count of ≤500/µl, regardless of the type of cancer, site of disease, or patients' gender, age, or tribe.

### Sample collection

About 20 ml of venous blood samples were collected into brain heart infusion broth and anaerobic broth under aseptic conditions. One set of two bottles was collected from each patient. Most of the patients received the first dose of antibiotics before sample collection.

### Sample processing

Aerobic and anaerobic blood culture bottles were incubated at 37 o C for up to seven days. The bottles were observed twice daily for turbidity, colour change, and haemolysis. Direct gram stain was done daily to detect bacterial growth. Regular subculture was done after one day of incubation, at 48 hours, and then at 96 hours. Each sample was sub cultured on blood agar, MacConkey agar, and chocolate blood agar (incubated at 5% CO2 candle jars) and incubated at 37°C for 48 h. If there was no broth change or negative subculture for up to seven days, blood culture was considered as negative.

Bacterial identification was done based on colonial morphology, gram stain, and standard biochemical tests 9.

The antimicrobial susceptibility of isolated organisms was detected by using the Kirby Bauer disc diffusion method according to the Clinical and Laboratory Standard Institute (CLSI) 9. Pure colonies of isolates were emulsified in distilled water to match the 0.5 McFarland turbidity standard. Antimicrobial discs were placed on the inoculated agar and incubated for 24 hours at 37 °C. Zones of inhibition were measured and determined as susceptible, intermediate, or resistant according to the Clinical and Laboratory Standard Institute guidelines. American Type Culture Collection (ATCC) isolates of Staphylococcus aureus (S. aureus), Escherichia coli (E. coli), Klebsiella pneumoniae (K. pneumoniae), and Pseudomonas aeruginosa (P. aeruginosa) were used as control strains. Gram-negative bacilli isolates were tested against amikacin, cefuroxime, Ceftazidime, ceftriaxone, ciprofloxacin, gentamicin, Meropenem, and Amoxicillin/clavulanic acid (HiMedia, India). The sensitivities of gram-positive cocci were tested against penicillin, tetracycline, erythromycin, clindamycin, oxacillin, gentamicin, and vancomycin (Hi-Media, India). Gram-negative isolates that showed resistance to third-generation cephalosporins were examined for extended-spectrum beta-lactamase (ESBL) production by double-disk synergy test. Methicillin-resistant S. aureus (MRSA) was detected using an oxacillin salt agar screening test 9.

### Data analysis

Data were analysed by statistical package for social science SPSS software version 24. A Chi-square test was used and P-value< 0.05 was considered significant in comparative data.

## Results

**Figure 1:**
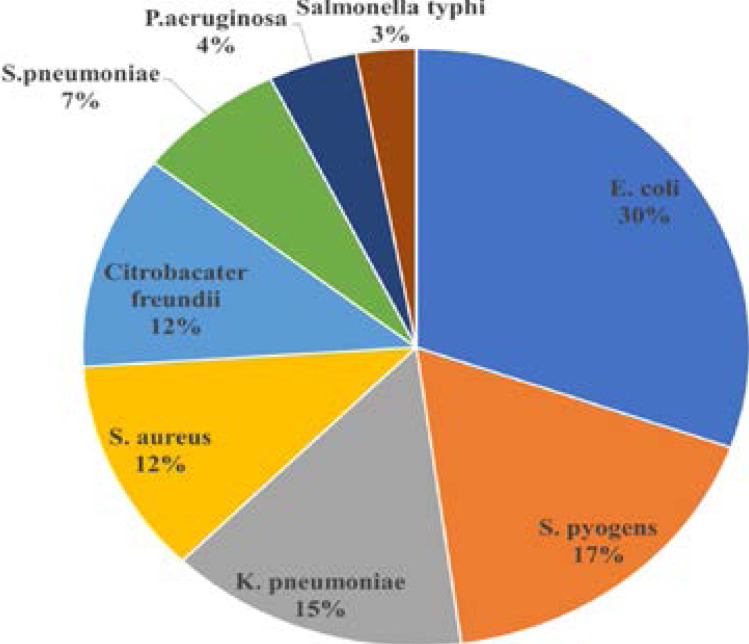
Distribution of Bacterial Isolates from the 69 Positive Blood Samples

A total of 69 (12%) out of 569 specimens were positive. Most of the isolates were gram negative bacilli (63.8%); *E. coli, K. pneumoniae, Citrobacter freundii, P. aeruginosa* and *Salmonella typhi*. Gram positive organisms represented 36.2% of the isolates, namely *Streptococcus pyogenes, S. aureus and Streptococcus pneumoniae*. Distribution of the isolated organisms is shown in Figure 1.

**Figure 2:**
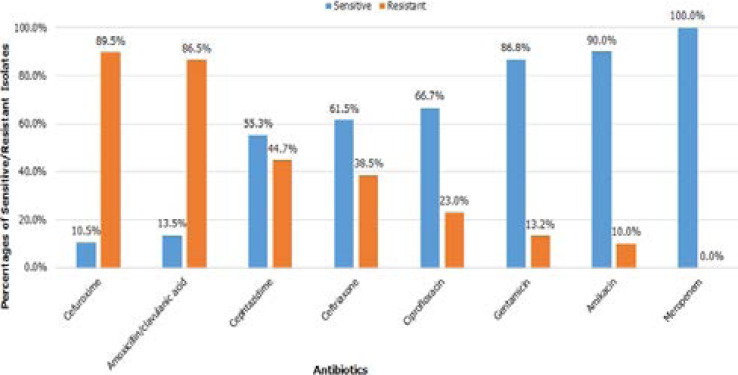
Antibiotic Susceptibility of the Gram-negative Isolates

No obligate anaerobic organism grew in the anaerobic bottles. Gram negative bacilli showed high levels of resistance to amoxicillin/clavulanic acid and to the cephalosporins. They were moderately sensitive to aminoglycosides and ciprofloxacin but were entirely sensitive to Meropenem (figure 2).

ESBL activity reached 34.1% among gram negative isolates. More than half (57%) of E. coli isolates were ESBL producers.

While all the gram-positive isolates were susceptible to vancomycin and gentamicin, high resistance rates were detected against tetracycline (60%), penicillin (44%) and erythromycin (40%). Sensitivity patterns of gram-positive organisms is shown in Table 1.

**Table 1:** Susceptibility Patterns of the Gram-positive Isolates

	Number and percentage of isolates resistant to antibiotics						
Organism	clindamycin	Penicillin	Vancomycin	Tetracycline	Erythromycin	Oxacillin	Gentamycin
**S.** *pyogenes* (n =12)	0/12 (0%)	0/12 (0%)	0/12 (0%)	7/12 (58.3%)	5/12 (41.6%)	0/12 (0%)	0/12 (0%)
*S.* *aureus* (n= 8)	3/8 (37.5%)	8/8 (100%)	0/8 (0%)	4/8 (50%)	4/8 (50%)	6/8 (75%)	0/8 (0%)
*S.* *pneumoniae* (n=5)	3/5 (60%)	3/5 (60%)	0/5 (0%)	4/5 (80%)	1/5 (20%)	-	-
Total resistance	6/25 (24%)	11/25 (44%)	0/25 (0%)	15/25 (60%)	10/25 (40%)	6/20 (30%)	0/20 (0%)

The majority (75%, n= 6/8) of *S. aureus* isolates were methicillin resistant staph aureus (MRSA).

## Discussion

BSI remains a common complication and one of the most important causes of morbidity and mortality among cancer patients 1. The nature and epidemiology of pathogens causing blood stream infections and their antibiotic susceptibility profiles in cancer patients is dynamic mandating implementing routine surveys to update the local antibiogram to guide appropriate selection of empiric antibiotic therapy 8.

In our study the rate of positive blood culture was 12%, This rate of growth is considerably low when compared to earlier studies in Sudan, Ethiopia, Malaysia and India, where the growth rates ranged between 35.2% and 69.4% 13–16. However, rates of 15% and 13.9% were reported by studies from India and Mexico respectively 7,17. Differences in detected growth rate might be due to differences in the characteristics of patients included in those studies (presence and absence of symptoms, neutrophils count, or type of tumor; hematologic malignancy versus solid tumors). In our case, in spite of collecting adequate volumes of blood and proper sampling time and technique, the low detection rate was a direct result of some patients may start empirical antibiotics therapy before samples collection or before arriving to hospital from health center.

Our study showed predominance of gram-negative pathogens in, 63.8%; mostly E. coli (30.4%) and K. *pneumoniae* (14.5%). These findings were reported by many similar studies in Egypt and Malaysia 7,15.

*S. aureus* represented only 11.6% of the isolates which contradicts the findings of a study conducted in our hospital in 2013, where *S. aureus* was the most frequent isolate (72%) 13.

We detected high levels of resistance in gram negative pathogens to antibiotics commonly used in our hospital. Our isolates showed alarmingly high rate of resistance to cefuroxime and amoxicillin/clavulanic acid (89.5% & 86.5% respectively) which can be explained by the irrational use and over prescription of these two drugs in the few last years.

Interestingly, higher rate of resistance to Ceftazidime (44.7%) was observed compared to ceftriaxone (38.5%). Increased utilization of Ceftazidime in our wards to treat Pseudomonad surgical wound infection may explain this finding.

On the other hand, aminoglycosides showed good sensitivity profile; gentamycin (86.8%) and amikacin (90%). Fortunately, Meropenem retained its activity against all gram-negative isolates, highlighting the efficacy of adopting the strategy of limiting the use of this broad-spectrum antibiotic as empiric therapy in our facility.

ESBL producing bacteria accounted for 34.1% of our gram-negative isolates compared to the results of a previous study carried in our hospital between 2010–2013, when the ESBL isolates reached 49.2% 19.

Many studies have reported ESBL producing Enterobacteriaceae between 11 and 45.4%, the variation in these results is directly related with antibiotic prescription policies 15–17.

Regarding antimicrobial resistant pattern of gram-positive pathogens, the highest rate of resistance was observed with tetracycline (60%), followed by penicillin (44%) and erythromycin (40%).

All the isolates were sensitive to gentamicin and vancomycin. While similar sensitivity patterns were observed in an Ethiopian study, different sensitivity profiles were reported in a study from another neighbour country, Ghana, where resistance rates to erythromycin and penicillin were low (8.82% and 27.7%) but vancomycin and gentamicin showed higher resistance rate compared to our setting (13.5 and 16.7%) 14,20. The percentage of MRSA isolates is notably higher in our hospital than in the Ethiopian and the Ghanaian studies, intensifying the need to intensify the infection control guidelines and to raise the knowledge of our staff about the impact of neglecting these measures.

## Conclusion

In conclusion, gram-negative bacilli with considerable multidrug resistance phenotypes were the dominant agents in BSI in cancer patients in Khartoum Oncology Hospital. Our findings highlight the importance of adopting antibiotics policies based on the local antibiotic susceptibility profiles. Our data also raise the need for continuous monitoring to ensure staff adherence to infection control measures in order to minimize the problem of multidrug resistance.

## What is already know on this topic

- Bloodstream infection is a major cause of morbidity and mortality in cancer patients.
- Using of broad-spectrum antibiotics had increased the problem of antibiotics resistance among the causative organisms of bloodstream infection in those patients.

## What this study adds

- The causative agents of bloodstream infection in our patients had shifted from gram positive to gram negative bacilli.
- Cephalosporins and penicillin, which are widely used empirically to treat bloodstream infection, are showing high rates of resistance.
